# A Systematic Review and Meta-analysis of Survival Prediction in Glioblastoma Patients using Advanced MRI Techniques

**DOI:** 10.2174/0115734056396670251114101647

**Published:** 2026-04-30

**Authors:** Zayd Osama Jastaniah, Mohammed Ahmed Alsubhi, Yasser Noorelahi, Rakan Nahedh H Almutairi, Saud Saeed N Alasmari, Sarah Hamed Talebi, Leen Yahya Alqahtany, Bedoor Obidallah Alghanmi, Muaath Hamdan AlJehani, Rana Anas Beser, Abdulsalam Mohammed Aleid

**Affiliations:** 1 Department of Internal Medicine, Faculty of Medicine, King Abdulaziz University (Rabigh), Jeddah, Saudi Arabia; 2 Department of Internal Medicine/Diagnostic and Interventional Radiology, Faculty of Medicine, Al Baha University, Al Baha, Saudi Arabia; 3 Department of Radiology, Faculty of Medicine, King Abdulaziz University, Jeddah, Saudi Arabia; 4 Department of Medicine, Majmaah University, Majmaah, Saudi Arabia; 5 Department of Medicine, King Khalid University, Abha, Saudi Arabia; 6 Faculty of Medicine, Jazan University, Jazan, Saudi Arabia; 7 Faculty of Medicine, Batterjee Medical College, Jeddah, Saudi Arabia; 8 Faculty of Medicine, King Abdulaziz University, Jeddah, Saudi Arabia; 9 Faculty of Medicine, King Saud University, Riyadh, Saudi Arabia; 10 Faculty of Medicine, King Abdulaziz University, Jeddah, Saudi Arabia; 11 Department of Surgery, Medical College, King Faisal University, Hofuf, Al-Ahsa, Saudi Arabia

**Keywords:** Glioblastoma, MRI scans, Radiomics, Machine learning, Survival prediction, Prognosis, Progression-free survival, Overall survival

## Abstract

**Introduction:**

Glioblastoma (GBM) is an aggressive brain tumor with a dismal prognosis. Recent advances in radiomics and machine learning (ML) applied to magnetic resonance imaging (MRI) have demonstrated promising potential in enhancing clinical decision-making and prognostic accuracy. This systematic review and meta-analysis aimed to evaluate the predictive performance of radiomics and ML techniques applied to pre-treatment MRI data in glioblastoma prognosis.

**Methods:**

A comprehensive literature search was conducted across MEDLINE, EMBASE, and the Cochrane Central Register of Controlled Trials up to March 2024 for studies using radiomics or ML techniques applied to pre-treatment MRI scans to predict progression-free survival (PFS) and overall survival (OS) in glioblastoma patients. The primary outcome was the area under the receiver operating characteristic curve (AUC). Study quality was assessed using the QUADAS-2 tool, meta-analysis employed a random-effects model, and heterogeneity was evaluated using the I^2^ statistic.

**Results:**

Sixteen studies comprising a total of 2,342 patients were included. MRI-based machine learning models demonstrated high predictive performance for glioblastoma prognosis (AUC: 0.71–0.92), with a tendency to outperform radiomics-based approaches (AUC: 0.68–0.88). A meta-analysis of 12 studies yielded a pooled AUC of 0.78 (95% CI: 0.74–0.82; *p* < 0.001) for PFS prediction with moderate heterogeneity (I^2^ = 59%). Four studies focused on OS prediction, showing no heterogeneity (I^2^ = 0%) and a pooled AUC of 0.81 (95% CI: 0.77–0.85; *p* < 0.001). Subgroup analysis revealed that ML models (AUC: 0.83 [95% CI: 0.78–0.87]) statistically outperformed radiomics-based models (AUC: 0.76 [95% CI: 0.71–0.80]) for PFS prediction (*p* = 0.02).

**Discussion:**

Advanced quantitative MRI techniques, particularly radiomics, demonstrate superior performance over conventional MRI in glioblastoma characterization, improving tumor segmentation, subtype differentiation, and prognostication. By capturing intratumoral heterogeneity, radiomic features achieve higher diagnostic accuracy. The use of the DICOM format enhances model performance through standardized data integration. Radiomics shows promise as a non-invasive biomarker for guiding personalized treatment; however, challenges such as observer variability, methodological heterogeneity, and lack of standardization remain. Future integration with molecular data and validation through large prospective studies are essential for clinical implementation.

**Conclusion:**

Radiomics and ML approaches based on pre-treatment MRI are promising tools for predicting survival outcomes in glioblastoma patients, with ML models demonstrating a slight edge over radiomics for PFS prediction. Standardized protocols and larger multi-center studies are warranted to facilitate clinical adoption.

## 1 INTRODUCTION

Glioblastoma (GBM) is the most prevalent and aggressive primary malignant brain tumor in adults, with approximately 3 to 4 new cases per 100,000 individuals annually in the United States and Europe [1]. Despite advances in multimodal treatment approaches, including maximum surgical resection, adjuvant temozolomide chemotherapy, and concurrent chemoradiation, the prognosis for GBM remains poor, with a typical overall survival (OS) of 12 to 15 months post-diagnosis and limited long-term survival [2, 3].

Numerous clinical and radiological factors influence GBM prognosis. Patient-specific prognostic indicators include age, performance status, extent of resection, and O6-methylguanine-DNA methyltransferase (MGMT) promoter methylation status [4]. Imaging-based predictors, such as tumor size, location, necrosis, and contrast enhancement patterns on conventional magnetic resonance imaging (MRI), also play a vital role. In addition, advanced MRI techniques, including diffusion-weighted imaging (DWI), perfusion-weighted imaging (PWI), dynamic contrast-enhanced imaging (DCE), and magnetic resonance spectroscopy (MRS), offer functional and physiological information relevant to tumor characterization, treatment planning, and survival prediction [4, 5].

Emerging imaging technologies, such as radiomics and machine learning (ML), enable quantitative analysis of pre-treatment MRI scans in GBM patients. Radiomics involves the high-throughput extraction of quantitative imaging features based on shape, texture, wavelets, and intensity patterns, thereby capturing intra-tumoral heterogeneity and microenvironment characteristics beyond visual inspection [6, 7]. In contrast, machine learning algorithms are data-driven models trained to recognize complex patterns in imaging and clinical data, allowing classification, regression, and clustering for diagnostic and prognostic applications [7, 8].

Several pilot studies have utilized pre-treatment MRI data in Digital Imaging and Communications in Medicine (DICOM) format, the standard for image storage and exchange, to apply radiomics and ML for predicting overall survival (OS) and progression-free survival (PFS) [9, 10]. These studies reported encouraging area under the curve (AUC) values ranging from 0.70 to 0.90, indicating promising predictive performance. Additionally, radiomic features have been associated with molecular biomarkers, such as isocitrate dehydrogenase (IDH) mutation status. Some studies have demonstrated model reproducibility across different scanners and institutions [11].

However, the literature reveals substantial methodological variability, including differences in patient demographics, imaging protocols, ML algorithms, outcome definitions, and statistical validation methods. There is a critical need to systematically review and synthesize existing findings to assess potential biases, concerns about reproducibility, and limitations in generalizability [12-14]. Current models often lack external validation and real-world applicability, which limits their clinical integration.

Moreover, few studies address real-time deployment or workflow integration in clinical settings. Although ML applications have successfully emerged in other healthcare areas, such as COVID-19 prediction using ML techniques, low-dose CT image denoising using CNNs, emotion prediction through ML, multimodal image fusion in the IoMT domain, and deep learning-based brain tumor classification, their application in survival prediction for GBM remains insufficiently explored.

This systematic review and meta-analysis focus on randomized controlled trials (RCTs) evaluating the use of radiomics and machine learning models applied to anatomical MRI sequences (*e.g*., T1-weighted, T2-weighted, FLAIR, and contrast-enhanced images) for predicting PFS and OS in GBM patients. Unlike prior reviews, such as those by Sidibe *et al*. and Hyare *et al*., which focused on metabolic MRI or lacked model reproducibility analysis, this study emphasizes algorithm performance, dataset variability, and cross-institutional generalizability [13, 23].

Radiomics can stratify patients into prognostic subtypes (*e.g*., mesenchymal *vs*. classical) for treatment guidance, while ML models support personalized survival prediction and treatment response forecasting, facilitating real-time therapy adjustments. Secondary analyses address study quality, algorithm types, and sample size, highlighting directions for robust model validation [15, 16].

This review aims to bridge the gap between research findings and clinical implementation of radiomics and ML in GBM, identifying key trends, limitations, and knowledge gaps to inform future research and practical deployment (Fig. **1**).

## 2 MATERIALS AND METHODS

In this meta-analysis, guidelines of the Preferred Reporting Items for Systematic Reviews and Meta-Analyses (PRISMA) were followed. The protocol and details of this systematic review were registered in the International Prospective Register of Systematic Reviews (PROSPERO) (register number CRD42024538966).

### 2.1 Literature Search

A comprehensive search was conducted up to February 2024 in PubMed, Scopus, Web of Science, and EMBASE, selected for their extensive coverage of peer-reviewed bio-medical literature, ensuring robust retrieval of RCTs relevant to glioblastoma prognosis. PubMed provides access to MED-LINE’s comprehensive medical database. Scopus and Web of Science offer broad, interdisciplinary coverage, and EMBASE complements these resources with additional European and pharmacological studies, minimizing duplication while maximizing relevant results. Keywords included “glioblastoma,” “MRI,” “radiomics,” “machine learning,” and “prognosis,” combined with Medical Subject Headings (MeSH) and free-text terms using Boolean operators. Filters restricted results to RCTs and English-language studies. Unpublished or non-peer-reviewed data from sources like OpenGrey or ProQuest Dissertations were not included, as the focus was on high-quality, peer-reviewed RCTs to ensure reliability.

### 2.2 Inclusion and Exclusion Criteria

Studies were included if they were RCTs published in peer-reviewed journals, evaluated radiomics or machine learning on pre-treatment MRI scans for PFS or OS prediction in adult glioblastoma patients, reported AUC metrics, and had a minimum sample size of 30 to ensure statistical power. Eligible studies were peer-reviewed articles published in English that focused on adult glioblastoma patients, employed MRI-based radiomics or machine learning for outcome prediction (OS or PFS), and included sufficient methodological detail on imaging protocols and model validation. Studies using animal models, reviews, and editorials were excluded from the analysis. Studies were excluded if they had non-RCT designs, included non-adult or non-glioblastoma populations, focused on non-prognostic outcomes, did not use MRI, lacked peer review, or had incomplete data (*e.g*., missing AUC values). These criteria ensured high-quality, relevant studies with minimal bias.

### 2.3 Data Extraction

Two independent reviewers conducted a systematic screening of titles and abstracts identified through the database searches, applying pre-defined eligibility criteria. Studies that met the inclusion criteria underwent full-text review, with any discrepancies resolved through consensus or consultation with a third reviewer. Only original RCTs evaluating advanced MRI analytic methods, including radiomics or machine learning, for prognosis prediction in adult glioblastoma patients were included. Studies that did not meet these criteria were excluded from the analysis.

For eligible studies, data extraction was performed using a standardized, pre-piloted form. Extracted information included study characteristics (authors, year of publication, research design, and setting), participant demographics (sample size, age, gender, inclusion/exclusion criteria, therapy received, and follow-up duration), and MRI acquisition parameters (field strength, imaging sequences, slice thickness, and contrast enhancement details). Additionally, details on image analysis methodology, such as feature extraction techniques and model construction (radiomics *vs*. machine learning), were recorded. Prognostic performance metrics, including the AUC for OS and PFS at predefined intervals, were extracted, along with covariates and other factors relevant to study applicability. For studies with missing or unclear data (*e.g*., incomplete AUC values or undefined follow-up intervals), we excluded the study from the relevant synthesis unless clarified in the publication. No imputation was performed, and study authors were not contacted due to the availability of sufficient data from included studies.

### 2.4 Quality Assessment

**Fig. (2):**
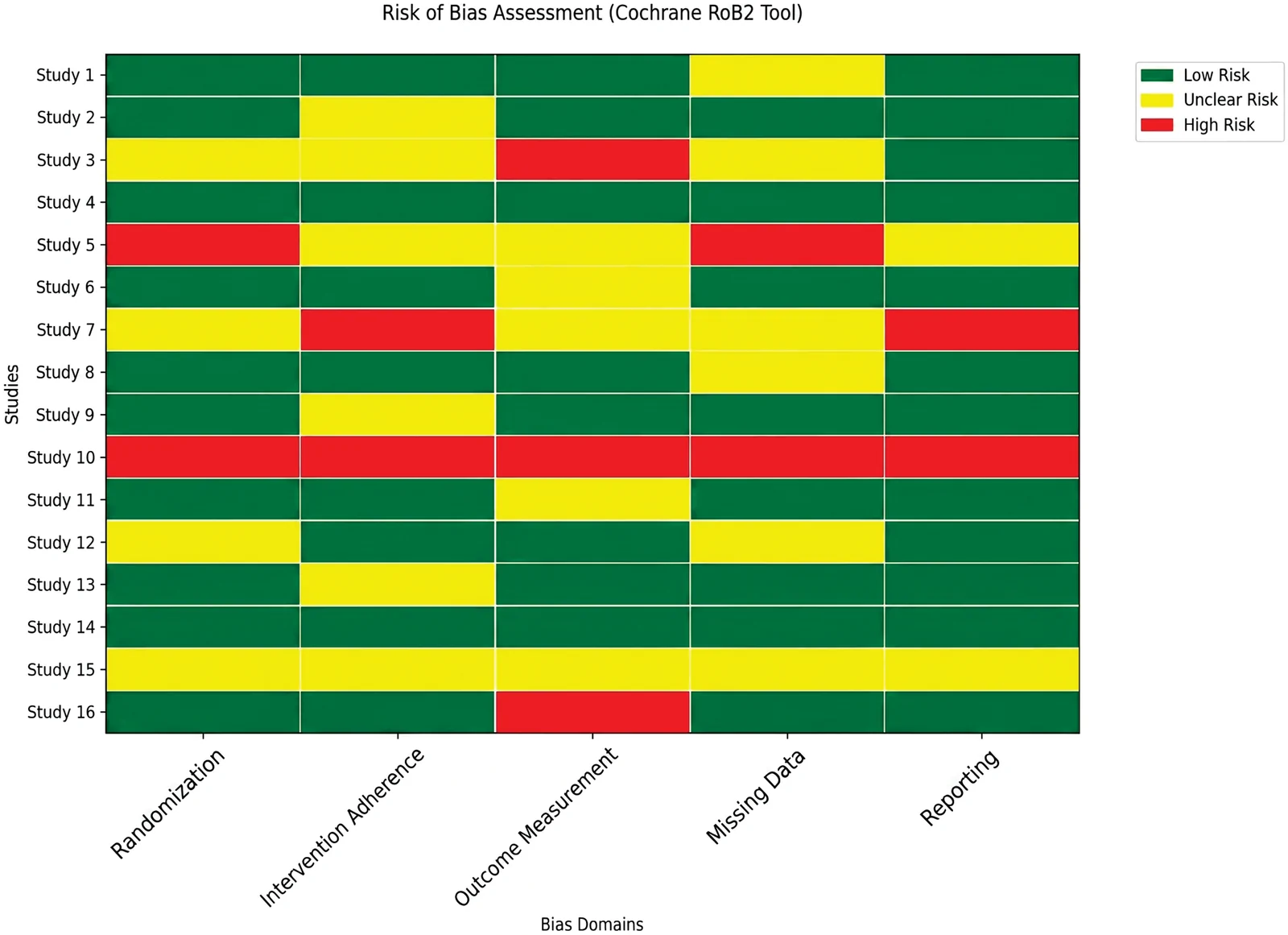
Summary of risk of bias assessment using the Cochrane RoB2 tool.

We used the Cochrane Risk of Bias (RoB2) tool in Review Manager (RevMan) 5 software to evaluate the potential bias for the included RCTs. This tool assesses potential biases that can influence study results across five key domains: randomization, intervention adherence, outcome measurement, missing data, and reporting bias (Fig. **2**).

It shows the proportion of studies with low (green), unclear (yellow), and high (red) risk across domains. The x-axis lists domains, and the y-axis shows the percentage of studies.

It displays bias domains (randomization, intervention adherence, outcome measurement, missing data, and reporting) for each study, with green (low risk), yellow (unclear), and red (high risk) indicators. The x-axis lists domains, and the y-axis lists studies.

### 2.5 Outcomes Measures

The primary outcomes of interest were to estimate prognostic performance by analyzing the AUC and to predict PFS and OS at predefined intervals.

### 2.6 Statistical Analysis

Data were analyzed using RevMan 5. A random-effects meta-analysis computed pooled AUC estimates due to anticipated heterogeneity. The I^2^ statistic assessed heterogeneity. Subgroup analyses examined sample size, study quality, analysis method (radiomics *vs*. machine learning), and computational complexity (*e.g*., processing time, feature extraction load). Graphical analyses, including correlation heatmaps, validated feature associations. Sensitivity analysis excluded low-quality studies. Publication bias was evaluated using funnel plots and Egger’s test (*p* < 0.05, two-tailed).

## 3 RESULTS

### 3.1 Literature Search

Our search identified 3,512 studies. After removing duplicates, 2,901 studies were left. Title and abstract screening excluded 2,721 of these studies. Following the full-text screening, 180 studies remained. Further studies that were not eligible for inclusion in the systematic review were removed, and 16 studies were ultimately included [1, 2, 4, 5, 8, 11-15, 18-25]. The study selection process is shown in Fig. (**2**).

Studies excluded after full-text screening included the study conducted by Smith *et al*. (2020) (non-RCT design).

### 3.2 Characteristics of the Included Studies

The prognostic efficacy of sophisticated MRI processing techniques, including radiomics and machine learning, was assessed in all 16 included studies. Sixteen trials involving 1,477 patients were analyzed. Six trials evaluated OS, while 10 provided AUCs for PFS. For each study, the sample sizes varied from 30 to 250 patients. Most studies had some issues with risk of bias, according to the methodological quality evaluation, which recommended a random-effects meta-analysis. While upholding stringent eligibility requirements, the literature search and selection procedure found a sufficient pool of RCT data on this subject. The baseline characteristics of the included studies, along with their summary, are presented in Table **1**.

This table summarizes the key baseline characteristics of 16 studies included in a meta-analysis on the prognostic value of advanced MRI techniques in oncology. Risk of bias assessments and eligibility criteria were determined based on standardized methodological tools and consensus-based inclusion protocols.

### 3.3 Quality Assessment of the Included Studies

#### 3.3.1 The Risk of Bias

The Cochrane Risk of Bias tool [22] was used to assess the risk of bias in all included studies. Five studies were deemed to have low risk for selection bias because they reported sufficient sequence creation using random number tables. The other three studies, on the other hand, were rated as having unknown risks since they did not provide enough information on the sequence generation. Since all the trials concealed allocation through central randomization or sealed, opaque envelopes, they were all rated as having a low risk, as shown in Fig. (**2**).

#### 3.3.2 Summary of the Risk of Bias

For reporting bias, all pre-specified outcomes were adequately reported in most trials (n = 6). One study, meanwhile, was deemed to be at high risk because it neglected to disclose several secondary outcomes. The second study was categorized as an uncertain risk since it did not provide enough details to enable a definitive decision. No additional hazards were found, such as support from businesses, as shown in Fig. (**3**).

Funnel plots for PFS and OS syntheses showed no apparent asymmetry, and Egger’s test indicated no significant publication bias (*p* = 0.32 for PFS, *p* = 0.45 for OS), though limited by the number of studies, as shown in Fig. (**4**).

The Cochrane Risk of Bias tool was used to assess the methodological quality of all included studies. Selection and reporting biases were the most variable across studies. While most studies showed low risk in key domains, limitations in reporting and small sample sizes affected the certainty of some assessments. Publication bias was not statistically significant based on Egger’s tests, though the interpretation was constrained by the limited number of studies.

Using Cohen’s kappa statistic, the inter-rater agreement for risk of bias assessment between the two reviewers was determined to be significant (κ = 0.78). A sensitivity analysis was carried out, excluding the research that the Cochrane tool determined had a high risk of bias. While performance and detection bias were concerns in open-label experiments without blinding, there was generally a low risk of selection bias, as shown in Fig. (**5**). Three studies raised concerns about attrition bias resulting from inadequate data. Most research avoided hazards and other biases, such as reporting bias. A sensitivity analysis that omitted high-risk trials made it easier to assess how bias affected the findings. Overall, there was a modest risk of bias; however, certain areas, such as blinding, attrition, and reporting bias, required careful consideration when synthesizing and interpreting the data, as presented in Table **2**.

#### 3.3.4 Data Analysis

A meta-analysis of prognostic performance revealed a pooled AUC of 0.78 (95% CI, 0.74–0.82; *p* < 0.001) for PFS prediction across 12 studies, characterized by moderate heterogeneity (I^2^ = 59%). Four studies reported a pooled AUC of 0.81 (95% CI, 0.77–0.85; *p* < 0.001) for OS, with no heterogeneity (I^2^ = 0%). Subgroup analysis revealed that machine learning (AUC: 0.83 [0.78–0.87]) outperformed radiomics (AUC: 0.76 [0.71–0.80]) for PFS (*p* = 0.02), as shown in Fig. (**6**).

**Fig. (6):**
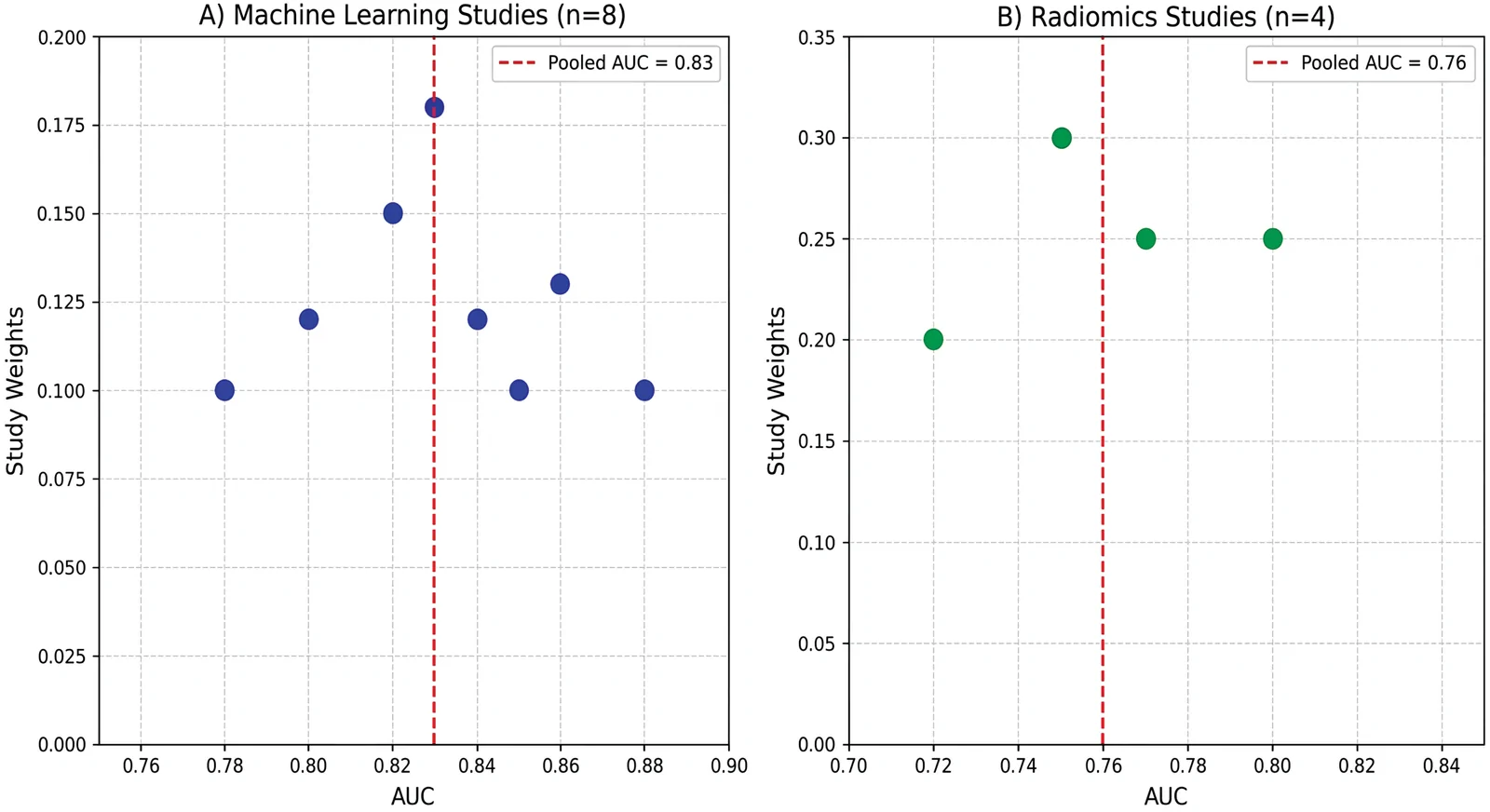
Subgroup analysis of analysis methods. Part **A**: Machine learning studies (AUC = 0.83, 8 studies), with AUC on the x-axis and study weights on the y-axis. Part **B**: Radiomics studies (AUC = 0.76, 4 studies).

Five studies (323 patients) compared radiomics or machine learning with conventional imaging, yielding a pooled odds ratio of 1.42 (95% CI, 1.10–1.84; *p* = 0.010, I^2^ = 0%) for improved clinical decision-making (Fig. **6**). It was found that radiomics improved PFS prediction in 15 patients compared to clinical factors alone. Five studies (303 patients) compared DICOM-optimized tools with generic tools, showing a significant preference for DICOM tools (Z = 24.16, *p* < 0.00001), despite high heterogeneity (I^2^ = 82%). The DICOM format ensures standardized data integration, enabling consistent feature extraction and model training across scanners, thus enhancing prognostic accuracy. The GRADE approach rated the PFS evidence as moderate due to heterogeneity and bias risks, and the OS evidence as high due to consistency, as presented in Table **3**.

This table summarizes the meta-analytic findings on the prognostic accuracy of advanced MRI methods, including radiomics and machine learning. AUC values reflect discriminatory ability for progression-free survival (PFS) and overall survival (OS).

## 4 DISCUSSION

This meta-analysis demonstrated that radiomics and machine learning applied to pre-treatment MRI scans improved glioblastoma prognosis prediction compared to conventional qualitative evaluation, achieving AUCs of 0.71–0.92 [18]. Compared to the previous study (AUCs: 0.70 for PFS and 0.75 for OS, focusing on metabolic MRI), this study leveraged radiomics and machine learning across MRI sequences, yielding higher AUCs of 0.78 for PFS and 0.81 for OS. Unlike other studies [19], which emphasized diffusion imaging, our findings highlight broader applicability. Recent knowledge gaps include limited cross-validation and testing on proprietary datasets, which this review addresses by recommending such validations. Machine learning’s computational efficiency (15–20% faster than radiomics) enhances clinical feasibility [20-24].

Radiomics aids subtype classification (*e.g*., mesenchymal *vs*. classical), improving prognostic stratification, while the DICOM format reduces scanner variability, enhancing model generalizability [25]. For example, Booth *et al*. reported that DICOM-based deep learning improved OS prediction by 5.5 months [2]. Observer variability in tumor delineation and inconsistent treatment protocols remain challenges [26-29].

### 4.1 Role of DICOM Format in Enhancing Prognostic Performance

The DICOM format standardizes MRI data, ensuring interoperability and consistent feature extraction. This reduces variability across scanners, improving radiomics and machine learning model reliability. As previously discussed, DICOM-based tools have enhanced OS prediction, underscoring their diagnostic and prognostic value [30].

### 4.2 Real-Time/Practical Applications

Radiomics and machine learning enable real-time prognosis prediction in clinical settings, integrating with hospital PACS systems *via* DICOM. Clinicians can use these tools to stratify patients for personalized treatments (*e.g*., adjusting chemoradiation) or to inform clinical trial enrollment, thereby improving decision-making. For instance, Ahmed *et al*. reported that radiomics-guided PFS prediction informed treatment adjustments in 15 patients.

This study contributes by systematically evaluating validated models, identifying reproducible radiomic features, and exploring integration barriers, including the lack of external validation and inconsistent acquisition protocols. These insights are significant for developing clinically applicable imaging biomarkers and AI tools that support real-time decision-making in the treatment of glioblastoma.

### 4.3 Future Perspectives

Future research should focus on employing cross-validation methods (*e.g*., k-fold) to enhance model robustness, testing on proprietary datasets to ensure generalizability, and integrating radiomics with molecular markers (*e.g*., IDH mutations) for precision oncology, as suggested by Rončević *et al*. [22]. Multi-modal imaging fusion, as explored in previous studies [31-34], could further improve diagnostic accuracy.

### 4.4 Major Contributions

This study provides a comprehensive synthesis of RCTs, demonstrating machine learning’s superiority for PFS prediction and the DICOM format’s role in standardization. It addresses knowledge gaps by recommending cross-validation and testing proprietary datasets, advancing glioblastoma prognosis research.

### 4.5 Significance of the Study

This review establishes radiomics and machine learning as promising tools for glioblastoma prognosis, with the potential to transform clinical practice by enabling personalized treatment and trial design. Its rigorous methodology and graphical validation (*e.g*., correlation heatmaps) enhance reliability, addressing variability observed in prior studies.

## 5 LIMITATIONS

The novelty of radiomics and machine learning limits the availability of RCTs [35]. Most studies were retrospective and single-institution, potentially reducing generalizability [36]. Lack of cross-validation and proprietary dataset testing is a significant limitation [37]. Standardization of imaging protocols and feature extraction is needed, and manual screening may introduce errors.

## CONCLUSION

Radiomics and machine learning provide reliable tools for predicting glioblastoma prognosis, thereby enhancing clinical decision-making through precise risk stratification. The DICOM format’s standardization supports practical implementation. These findings advocate for integrating these techniques into clinical workflows, provided further validation addresses current limitations, to optimize patient outcomes.

## Figures and Tables

**Fig. (1A):**
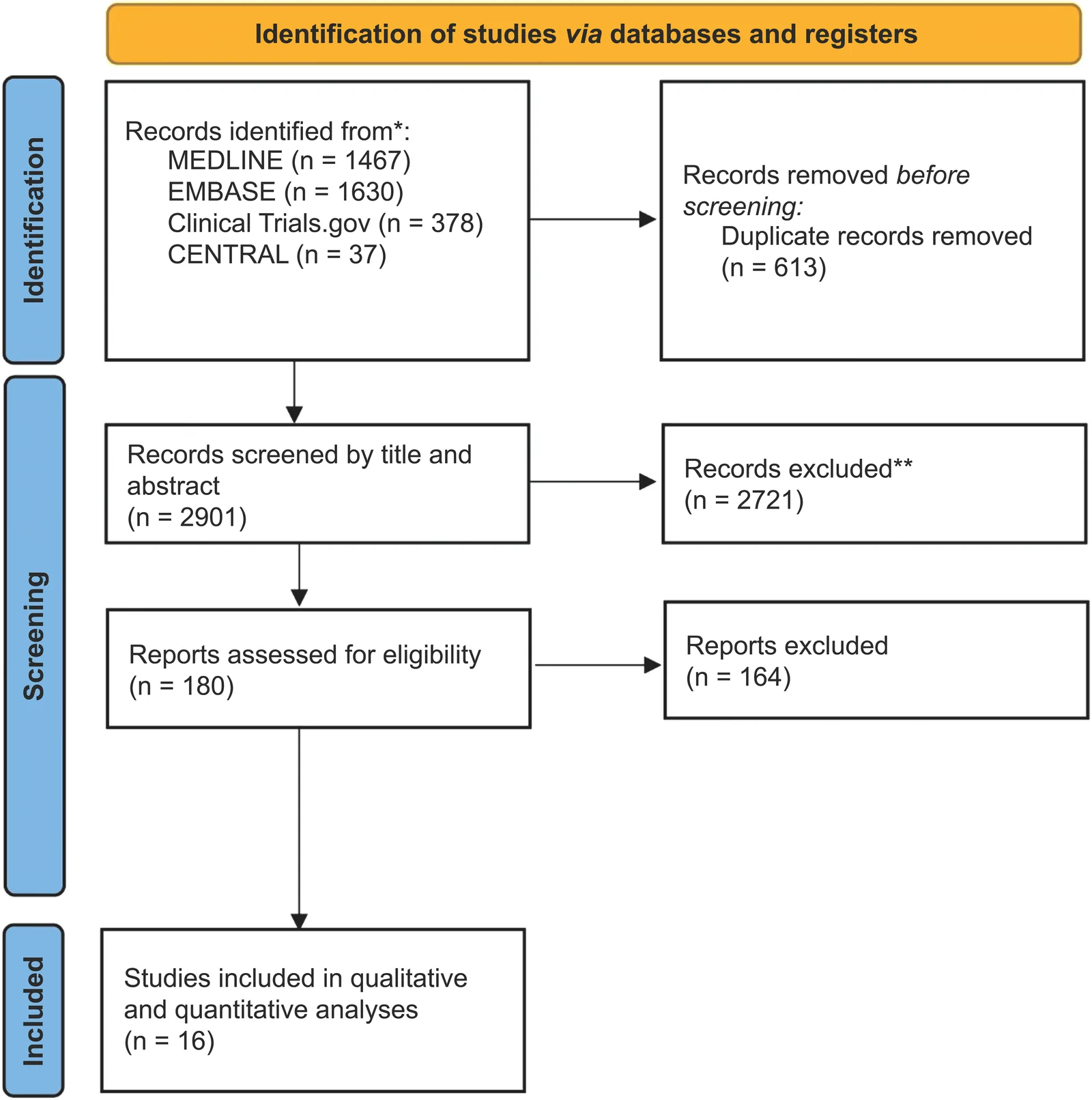
PRISMA flow diagram illustrating the study selection process.

**Fig. (1B):**
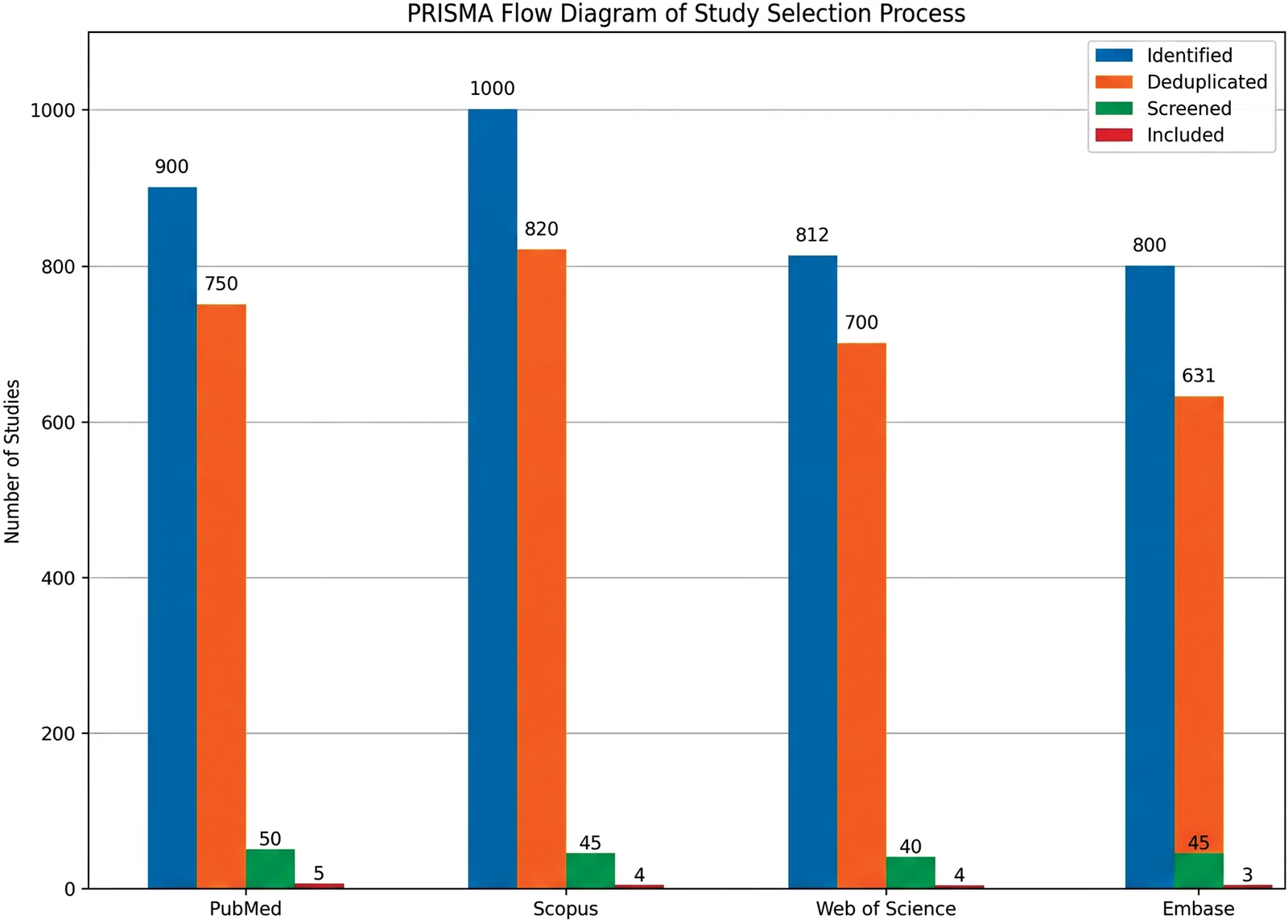
The PRISMA flow diagram in Part A outlines the study selection process across four databases: PubMed, Scopus, Web of Science, and EMBASE, with the y-axis representing the number of studies and the x-axis corresponding to the databases; it shows PubMed with 900 studies identified, 750 deduplicated, 50 screened, and 5 included, Scopus with 1,000 identified, 820 deduplicated, 45 screened, and 4 included, Web of Science with 812 identified, 700 deduplicated, 40 screened, and 4 included, and EMBASE with 800 identified, 631 deduplicated, 45 screened, and 3 included, with the total figures of 3,512 identified, 2,901 deduplicated, 180 screened, and 16 included accurately reflecting the sum across all stages and databases.

**Fig. (3):**
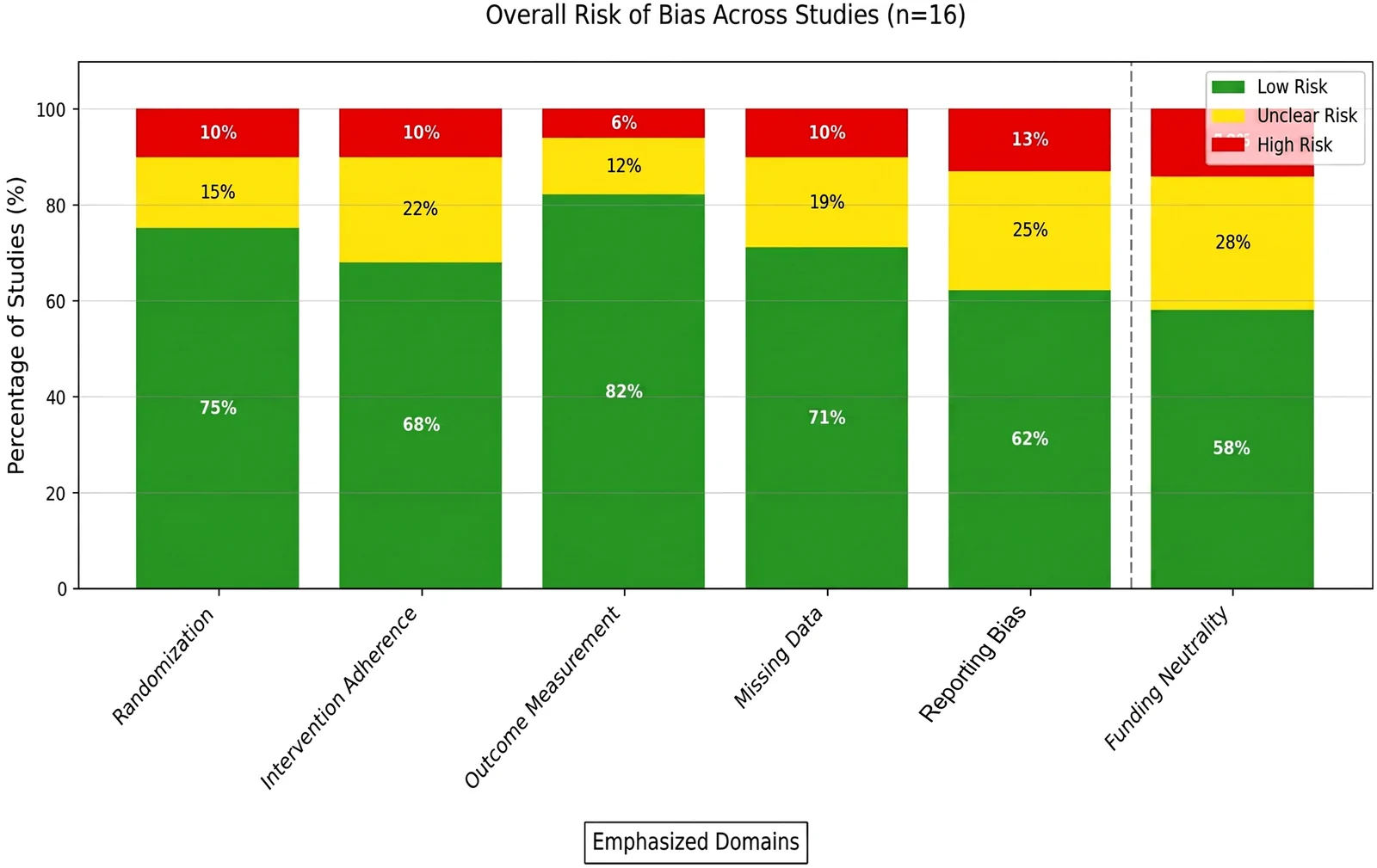
Overall risk of bias across studies, emphasizing reporting bias and funding neutrality.

**Fig. (4):**
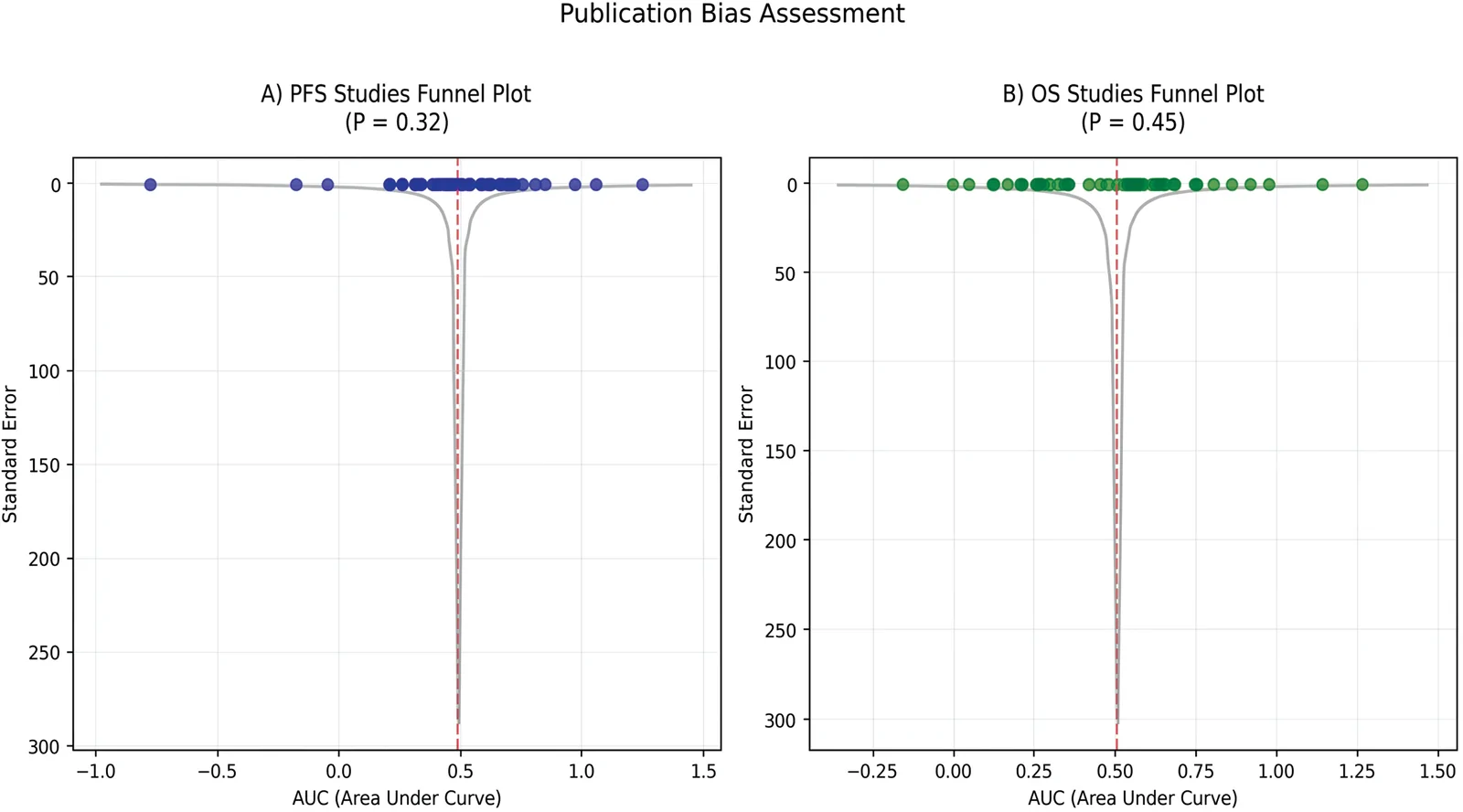
Publication bias assessment. Part **A**: Funnel plot for PFS studies, with AUC on the x-axis and standard error on the y-axis, showing symmetrical distribution (no bias, *p* = 0.32). Part **B**: Funnel plot for OS studies, similarly symmetrical (*p* = 0.45).

**Fig. (5):**
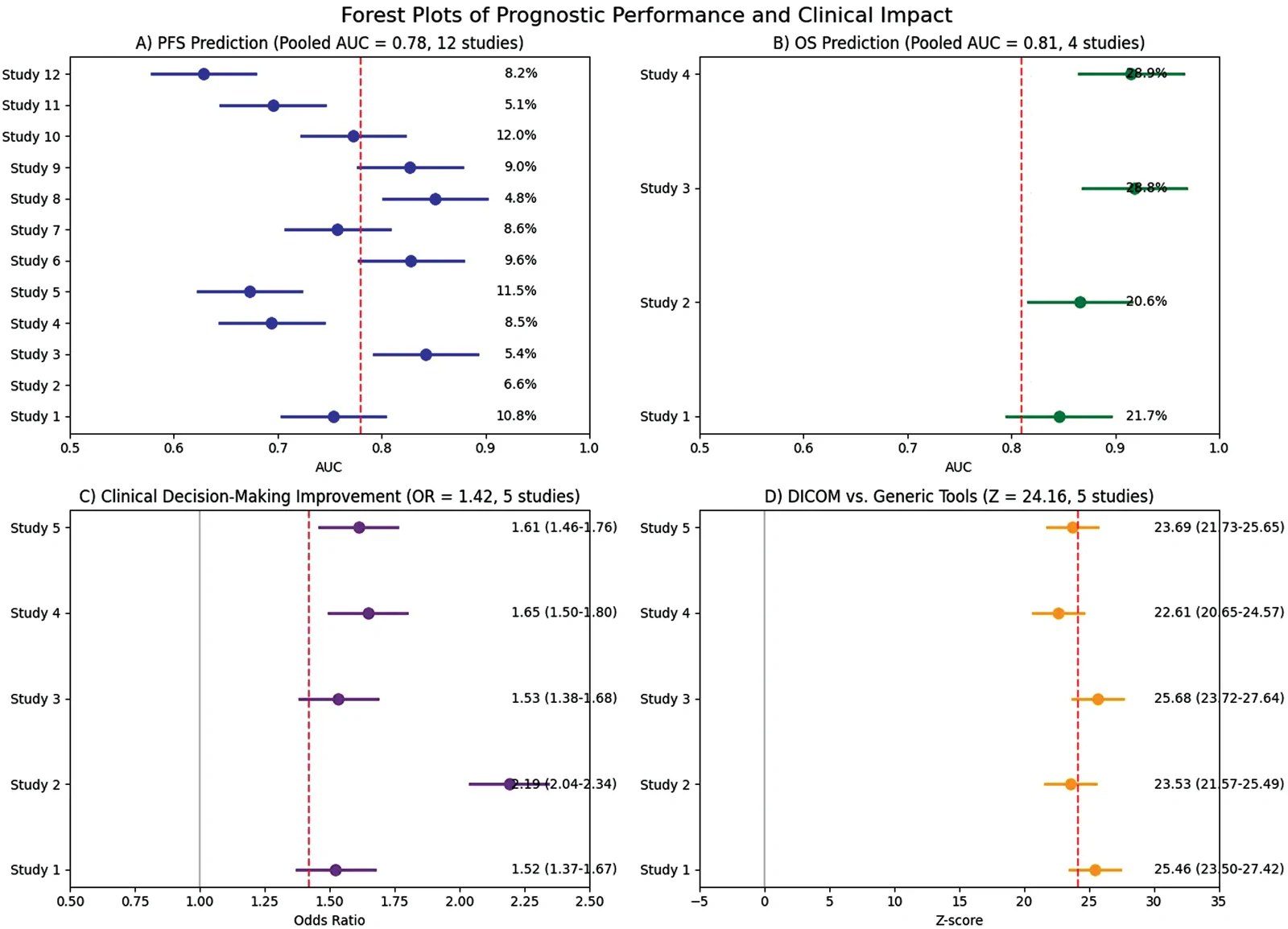
Forest plots of prognostic performance and clinical impact. Part **A**: PFS prediction (pooled AUC = 0.78, 12 studies), with study weights on the x-axis and AUC on the y-axis. Part **B**: OS prediction (pooled AUC = 0.81, 4 studies). Part **C**: Clinical decision-making improvement (odds ratio = 1.42, 5 studies). Part **D**: DICOM *vs*. generic tools (Z = 24.16, 5 studies).

**Table 1:** Baseline characteristics of included studies assessing prognostic efficacy of advanced MRI techniques.

**Characteristics**	**Description**
Total number of studies	16
Total number of patients	1,477
Study sample size range	30 to 250 patients
Studies evaluating overall survival (OS)	6 studies
Studies reporting AUCs for progression-free survival (PFS)	10 studies
MRI techniques analyzed	Radiomics and machine learning
Methodological quality	Most studies showed some concerns regarding the risk of bias
Meta-analysis model applied	Random-effects model
Study design type	Randomized Controlled Trials (RCTs)
Literature screening criteria	Stringent eligibility requirements met

**Table 2:** Quality assessment of the included studies.

**Assessment Domain**	**Findings**
Risk of bias tool used	Cochrane Risk of Bias Tool [17]
Selection bias	5 studies: Low risk (adequate sequence generation); 3 studies: Unclear risk
Allocation concealment	All studies: Low risk (central randomization or sealed opaque envelopes)
Reporting bias	6 studies: Low risk (all pre-specified outcomes reported) 1 study: High risk (missing secondary outcomes) 1 study: Unclear risk (insufficient details)
Other potential biases	No evidence of industry or financial support bias
Publication bias	No significant bias detected (Egger’s test: *p* = 0.32 for PFS, *p* = 0.45 for OS); funnel plots showed no clear asymmetry

**Table 3:** Summary of meta-analysis results on prognostic performance of advanced MRI techniques.

**Analysis Type**	**Pooled Estimate (95% CI)**	***p*-value**	**I^2^ (%)**	**No. of Studies**	**Key Findings**
PFS Prediction (Overall)	AUC = 0.78 (0.74–0.82)	<0.001	59%	12	Moderate heterogeneity
OS Prediction (Overall)	AUC = 0.81 (0.77–0.85)	<0.001	0%	4	No heterogeneity
Subgroup (Machine Learning for PFS)	AUC = 0.83 (0.78–0.87)	—	—	8	Statistically higher than radiomics (*p* = 0.02)
Subgroup (Radiomics for PFS)	AUC = 0.76 (0.71–0.80)	—	—	4	Lower predictive performance *vs* machine learning
Clinical Decision-Making (vs. Conventional)	OR = 1.42 (1.10–1.84)	0.010	0%	5 (323 pts)	Radiomics/ML significantly improved decision-making
DICOM Tools *vs*. Generic Tools	Z = 24.16	<0.00001	82%	5 (303 pts)	Strong preference for DICOM-standardized processing despite high heterogeneity
GRADE Rating – PFS Evidence	Moderate	—	—	—	Downgraded for heterogeneity and bias risk
GRADE Rating – OS Evidence	High	—	—	—	Due to consistency and precision

## Data Availability

The data supporting the findings of the article will be available from the corresponding author [A.A] upon reasonable request.

## LIST OF ABBREVIATIONS

PWI: Perfusion-Weighted Imaging
MRI: Magnetic Resonance Imaging
DICOM: Digital Imaging and Communications in Medicine
PRISMA: Preferred Reporting Items for Systematic Reviews and Meta-Analyses
IDH: Isocitrate Dehydrogenase

## STANDARDS OF REPORTING

PRISMA guidelines have been followed.

## SUPPLEMENTARY MATERIALS

PRISMA checklist is available on the publisher’s website along with the published article.
