# Supplementary material for: A Systematic Review and Meta-analysis of Survival Prediction in Glioblastoma Patients using Advanced MRI Techniques
Source: Curr Med Imaging. 2026 Apr 30;22:E15734056396670. doi: 10.2174/0115734056396670251114101647 (PMC13575947; doi:10.2174/0115734056396670251114101647)
Supplement: Supplementary file 1 [file CMIM-22-E15734056396670_SD1.pdf]

1875-6603/26 Send Orders for Reprints to reprints@benthamscience.net



BENTHAM SCIENCE

CrossMark

Current Medical Imaging

Content list available at: https://benthamscience.com/journals/cmir

**Supplementary Material**

# A Systematic Review and Meta-Analysis of Survival Prediction in Glioblastoma Patients Using Advanced MRI Techniques

Zayd Osama Jastaniah[1], Mohammed Ahmed Alsubhi[2], Yasser Noorelahi[3], Rakan Nahedh H Almutairi[4], Saud Saeed N Alasmari[5], Sarah Hamed Talebi[6], Leen Yahya Alqahtany[7], Bedoor Obidallah Alghanmi[8], Muaath Hamdan AlJehani[9], Rana Anas Beser[10] and Abdulsalam Mohammed Aleid[11,*]

[1]*Department of Internal Medicine, Faculty of Medicine, King Abdulaziz University (Rabigh), Jeddah, Saudi Arabia, zjastaniah@kau.edu.sa*
[2]*Department of Internal Medicine/Diagnostic and Interventional Radiology, Faculty of Medicine, Al Baha University, Al Baha, Saudi Arabia*
[3]*Department of Radiology, Faculty of Medicine, King Abdulaziz University, Jeddah, Saudi Arabia*
[4]*Department of Medicine, Majmaah University, Majmaah, Saudi Arabia*
[5]*Department of Medicine, King Khalid University, Abha, Saudi Arabia*
[6]*Faculty of Medicine, Jazan University, Jazan, Saudi Arabia*
[7]*Faculty of Medicine, Batterjee Medical College, Jeddah, Saudi Arabia*
[8]*Faculty of Medicine, King Abdulaziz University, Jeddah, Saudi Arabia*
[9]*Faculty of Medicine, King Saud University, Riyadh, Saudi Arabia*
[10]*Faculty of Medicine, King Abdulaziz University, Jeddah, Saudi Arabia*
[11]*Department of Surgery, Medical College, King Faisal University, Hofuf, Al-Ahsa, Saudi Arabia*

**PRISMA 2020 checklist for systematic review and meta-analysis of survival prediction in glioblastoma using advanced MRI techniques.**

| Section and Topic | Item # | Checklist Item | Location Where Item is Reported |
|---|---|---|---|
| **TITLE** | - | - | - |
| Title | 1 | Identify the report as a systematic review. | Page 1: “A Systematic Review and Meta-Analysis of Survival Prediction in Glioblastoma Patients Using Advanced MRI Techniques” |
| **ABSTRACT** | - | - | - |
| Abstract | 2 | See the PRISMA 2020 for Abstracts checklist. | Page 1: Abstract summarizes background, methods, results, and conclusions, including GRADE certainty (moderate for PFS, high for OS). |
| **INTRODUCTION** | - | - | - |
| Rationale | 3 | Describe the rationale for the review in the context of existing knowledge. | Pages 2-3: Discusses glioblastoma prognosis, advances in MRI techniques, variability in studies, and need for comprehensive review. |
| Objectives | 4 | Provide an explicit statement of the objective(s) or question(s) the review addresses. | Page 3: Aims to synthesize RCT data on prognostic performance of quantitative MRI for OS and PFS, with secondary analysis on study quality and algorithm type. |
| **METHODS** | - | - | - |

DOI: 10.2174/0115734056396670251114101647, 2026, *22*, e15734056396670

| Section and Topic | Item # | Checklist Item | Location Where Item is Reported |
|---|---|---|---|
| Eligibility criteria | 5 | Specify the inclusion and exclusion criteria for the review and how studies were grouped for the syntheses. | Pages 4-5: Inclusion: RCTs, adult glioblastoma patients, advanced MRI analytics, prognosis prediction. Exclusion: non-RCTs, non-adult/glioblastoma populations, non-prognostic outcomes, non-MRI studies. Grouped by radiomics *vs*. machine learning. |
| Information sources | 6 | Specify all databases, registers, websites, organisations, reference lists and other sources searched or consulted to identify studies. Specify the date when each source was last searched or consulted. | Page 4: Searched MEDLINE, EMBASE (*via* Ovid), Cochrane Central Register of Controlled Trials, ClinicalTrials.gov, up to February 2024. |
| Search strategy | 7 | Present the full search strategies for all databases, registers and websites, including any filters and limits used. | Page 4, Supplementary Table S1: Full MEDLINE search strategy provided; adapted for other databases. |
| Selection process | 8 | Specify the methods used to decide whether a study met the inclusion criteria of the review, including how many reviewers screened each record and each report retrieved, whether they worked independently, and if applicable, details of automation tools used in the process. | Page 5: Two independent reviewers screened titles/abstracts and full texts, with discrepancies resolved by consensus or third reviewer. No automation tools used. |
| Data collection process | 9 | Specify the methods used to collect data from reports, including how many reviewers collected data from each report, whether they worked independently, any processes for obtaining or confirming data from study investigators, and if applicable, details of automation tools used in the process. | Page 5: Two reviewers independently extracted data using a standardized form; no investigator contact or automation tools used. |
| Data items | 10a | List and define all outcomes for which data were sought. Specify whether all results that were compatible with each outcome domain in each study were sought, and if not, the methods used to decide which results to collect. | Page 5: Primary outcomes: AUC for PFS and OS at predefined intervals. All relevant AUC results sought. |

| Section and Topic | Item # | Checklist Item | Location Where Item is Reported |
|---|---|---|---|
| | 10b | List and define all other variables for which data were sought. Describe any assumptions made about any missing or unclear information. | Page 5: Extracted study characteristics, participant demographics, MRI parameters, image analysis methods. Missing data handled by exclusion from synthesis. |
| Study risk of bias assessment | 11 | Specify the methods used to assess risk of bias in the included studies, including details of the tool(s) used, how many reviewers assessed each study and whether they worked independently, and if applicable, details of automation tools used in the process. | Page 5: Cochrane RoB2 tool in RevMan 5, assessing five domains. Two reviewers assessed independently (Cohen's kappa = 0.78). |
| Effect measures | 12 | Specify for each outcome the effect measure(s) used in the synthesis or presentation of results. | Page 6: AUC for PFS and OS; odds ratios for efficacy and clinical decision-making analyses. |
| Synthesis methods | 13a | Describe the processes used to decide which studies were eligible for each synthesis. | Page 6: Studies grouped by outcome (PFS, OS) and analysis method (radiomics *vs*. machine learning) based on eligibility and data availability (Table 1). |
| | 13b | Describe any methods required to prepare the data for presentation or synthesis, such as handling of missing summary statistics, or data conversions. | Page 6: Missing AUC values excluded from meta-analysis; no conversions needed. |
| | 13c | Describe any methods used to tabulate or visually display results of individual studies and syntheses. | Page 6: Results in Tables 1-4; forest plots in Figures 4-6. |
| | 13d | Describe any methods used to synthesize results and provide a rationale for the choice(s). If meta-analysis was performed, describe the model(s), method(s) to identify the presence and extent of statistical heterogeneity, and software package(s) used. | Page 6: Random-effects meta-analysis due to heterogeneity, I2 for heterogeneity, RevMan 5 software. |
| | 13e | Describe any methods used to explore possible causes of heterogeneity among study results. | Page 6: Subgroup analyses by sample size, study quality, and image analysis approach. |
| | 13f | Describe any sensitivity analyses conducted to assess robustness of the synthesized results. | Page 6: Sensitivity analysis excluded high-risk bias studies. |

| Section and Topic | Item # | Checklist Item | Location Where Item is Reported |
|---|---|---|---|
| Reporting bias assessment | 14 | Describe any methods used to assess risk of bias due to missing results in a synthesis. | Page 6: Funnel plots and Egger's test used when sufficient studies available. |
| Certainty assessment | 15 | Describe any methods used to assess certainty (or confidence) in the body of evidence for an outcome. | Page 7: GRADE approach applied for PFS and OS outcomes. |
| **RESULTS** | - | - | - |
| Study selection | 16a | Describe the results of the search and selection process, from the number of records identified in the search to the number of studies included in the review, ideally using a flow diagram. | Page 6: 3,512 studies identified, 2,901 after deduplication, 2,721 excluded after title/abstract screening, 180 full-text screened, 16 included. Flow diagram in Figure 1. |
| | 16b | Cite studies that might appear to meet the inclusion criteria, but which were excluded, and explain why they were excluded. | Page 6, Supplementary Table S2: Key excluded studies cited with reasons (*e.g*., non-RCT, pediatric population). |
| Study characteristics | 17 | Cite each included study and present its characteristics. | Page 6: 16 studies cited [1, 2, 4, 5, 8, 11-15, 18-25]; characteristics in Table 1. |
| Risk of bias in studies | 18 | Present assessments of risk of bias for each included study. | Pages 6-7: Cochrane RoB2 results, Figures 2-3; 5 studies low risk for selection bias, 3 with attrition bias. |
| Results of individual studies | 19 | For all outcomes, present, for each study: (a) summary statistics for each group and (b) an effect estimate and its precision, ideally using structured tables or plots. | Pages 6-7: AUC values, odds ratios in Tables 2-4, forest plots in Figures 4-6. |
| Results of syntheses | 20a | For each synthesis, briefly summarise the characteristics and risk of bias among contributing studies. | Page 6: 12 studies for PFS (I2 = 59%), 4 for OS (I2 = 0%); risk of bias summarized. |
| | 20b | Present results of all statistical syntheses conducted. If meta-analysis was done, present for each the summary estimate and its precision and measures of statistical heterogeneity. | Page 6: Pooled AUC: 0.78 (95% CI 0.74–0.82, I2 = 59%) for PFS; 0.81 (95% CI 0.77–0.85, I2 = 0%) for OS. Odds ratios in Tables 2-4. |
| | 20c | Present results of all investigations of possible causes of heterogeneity among study results. | Page 6: Subgroup analysis showed machine learning outperformed radiomics for PFS (P = 0.02). |
| | 20d | Present results of all sensitivity analyses conducted to assess the robustness of the synthesized results. | Page 6: Sensitivity analysis excluding high-risk bias studies supported main findings. |

| Section and Topic | Item # | Checklist Item | Location Where Item is Reported |
|---|---|---|---|
| Reporting biases | 21 | Present assessments of risk of bias due to missing results for each synthesis assessed. | Page 7: Funnel plots and Egger's test (P = 0.32 for PFS, P = 0.45 for OS) showed no significant publication bias. |
| Certainty of evidence | 22 | Present assessments of certainty (or confidence) in the body of evidence for each outcome assessed. | Page 7: GRADE results for PFS (moderate) and OS (high) presented. |
| **DISCUSSION** | - | - | - |
| Discussion | 23a | Provide a general interpretation of the results in the context of other evidence. | Pages 8-9: Radiomics and machine learning outperform traditional MRI; aligns with prior studies. |
| | 23b | Discuss any limitations of the evidence included in the review. | Page 9: Limited studies, retrospective data, lack of standardization. |
| | 23c | Discuss any limitations of the review processes used. | Page 9: Reliance on published data, potential oversight of non-English studies, manual screening. |
| | 23d | Discuss implications of the results for practice, policy, and future research. | Pages 8-9: Clinical potential, need for standardization, larger prospective studies. |
| **OTHER INFORMATION** | - | - | - |
| Registration and protocol | 24a | Provide registration information for the review, including register name and registration number, or state that the review was not registered. | Page 4: Registered in PROSPERO (CRD42024538966). |
| | 24b | Indicate where the review protocol can be accessed, or state that a protocol was not prepared. | Page 4: Protocol in PROSPERO (https://www.crd.york.ac.uk/prospero/display_record.php?RecordID=CRD42024538966). |
| | 24c | Describe and explain any amendments to information provided at registration or in the protocol. | Page 4: No protocol amendments made. |
| Support | 25 | Describe sources of financial or non-financial support for the review, and the role of the funders or sponsors in the review. | Page 10: Funded by Deanship of Scientific Research, King Faisal University [Grant No. KFU250580]. |
| Competing interests | 26 | Declare any competing interests of review authors. | Page 10: No conflicts of interest declared. |

| Section and Topic | Item # | Checklist Item | Location Where Item is Reported |
|---|---|---|---|
| Availability of data, code and other materials | 27 | Report which of the following are publicly available and where they can be found: template data collection forms; data extracted from included studies; data used for all analyses; analytic code; any other materials used in the review. | Page 10, Supplementary Table S3: Data collection form and extracted data available; no analytic code used. |

© 2026 The Author(s). Published by Bentham Science Publisher.

This is an open access article distributed under the terms of the Creative Commons Attribution 4.0 International Public License (CC-BY 4.0), a copy of which is available at: https://creativecommons.org/licenses/by/4.0/legalcode. This license permits unrestricted use, distribution, and reproduction in any medium, provided the original author and source are credited.

**HOW TO CITE:**
Jastaniah Z, Alsubhi M, Noorelahi Y, Almutairi R, Alasmari S, Talebi S, Alqahtany L, Alghanmi B, AlJehani M, Beser R, Aleid A. A Systematic Review and Meta-analysis of Survival Prediction in Glioblastoma Patients using Advanced MRI Techniques. Curr Med Imaging, 2026; 22: e15734056396670.
http://dx.doi.org/10.2174/0115734056396670251114101647
